# Familial translocation between chromosomes 3 and 10: meiotic segregation, diagnostics and clinical features of chromosomal imbalance

**DOI:** 10.18699/vjgb-25-72

**Published:** 2025-09

**Authors:** A.V. Vozilova, A.S. Tarasova, E.A. Ivanov, V.P. Pushkarev, N.I. Nalyotova, A.I. Pobedinskaya, A.S. Sabitova, N.V. Shilova

**Affiliations:** Chelyabinsk Regional Children’s Clinical Hospital, Chelyabinsk, Russia; Chelyabinsk Regional Children’s Clinical Hospital, Chelyabinsk, Russia; Chelyabinsk Regional Children’s Clinical Hospital, Chelyabinsk, Russia; Chelyabinsk Regional Children’s Clinical Hospital, Chelyabinsk, Russia; Chelyabinsk Regional Children’s Clinical Hospital, Chelyabinsk, Russia; Chelyabinsk Regional Children’s Clinical Hospital, Chelyabinsk, Russia; Chelyabinsk Regional Children’s Clinical Hospital, Chelyabinsk, Russia; Bochkov Research Centre for Medical Genetics, Moscow, Russia

**Keywords:** reciprocal translocations, meiotic segregation, genome imbalance, der(3), der(10), GTG-banded chromosomes, clinical exome sequencing, chromosomal microarray, 3p deletion syndrome, реципрокная транслокация, мейотическая сегрегация, хромосомный дисбаланс, дериватная хромосома 3, дериватная хромосома 10, GTG-окрашивание хромосом, клиническое секвенирование экзома, хромосомный микроматричный анализ, 3р делеционный синдром

## Abstract

Reciprocal translocations are the most common structural chromosomal rearrangements, occurring at a frequency of 0.08–0.3 % in the human population. The vast majority of carriers of reciprocal translocations are phenotypically normal, but have an increased risk of miscarriage or the birth of children with intellectual disabilities and multiple congenital abnormalities due to meiotic malsegregation of chromosomes involved in the translocation. This study presents a familial case of translocation involving the distal regions of the short arms of chromosomes 3 and 10, detected in seven family members across three generations. The investigation was prompted by the detection of a deletion 10p15 and a duplication 3p25 revealed through clinical exome sequencing in a proband exhibiting phenotypic abnormalities, which may correspond to der(10)t(3;10)(p25;p15). GTG cytogenetic study of the proband’s family revealed that the mother, grandmother, aunt and brother – none of whom displayed any clinical or phenotypic manifestations – were carriers of a balanced chromosomal rearrangement, t(3;10)(p25;p15). By contrast, the karyotype of the proband’s sibling – a girl with severe cognitive, neurological, and developmental abnormalities – was found to be 46,XX,der(3)t(3;10)(p25;p15)dmat. Molecular karyotyping facilitated further clarification of the chromosomal imbalance and the precise breakpoints on both chromosomes involved in the translocation. This study provides a detailed description of the clinical and phenotypic manifestations resulting from the presence of derivative chromosomes 3 and 10 in the karyotype. Additionally, it discusses the mechanisms underlying the formation of chromosomal imbalances in the family members with the abnormal phenotype, the relationship between the severity of clinical manifestations and changes in gene dosage due to chromosomal rearrangements, as well as potential preventive and rehabilitative measures aimed at reducing the risk of chromosomal pathology in the families with carriers of autosomal reciprocal translocations.

## Abstract (ru)

Реципрокные транслокации являются наиболее частой структурной хромосомной перестройкой и встречаются в популяции с частотой 0.08–0.3 %. Большинство носителей реципрокных транслокаций фенотипически нормальны, но имеют повышенный риск привычного невынашивания беременности или рождения детей с нарушением интеллектуального развития и множественными врожденными аномалиями и/или пороками развития вследствие патологической мейотической сегрегации хромосом, вовлеченных в транслокацию. В данной работе представлен семейный случай транслокации между дистальными участками короткого плеча хромосомы 3 и короткого плеча хромосомы 10, когда перестройка между хромосомами 3 и 10 была обнаружена у семи членов семьи в трех поколениях. Поводом для обследования семьи стало выявление при клиническом секвенировании экзома делеции сегмента p15 хромосомы 10 и дупликации сегмента p25 хромосомы 3 у пробанда с аномалиями фенотипа, что может соответствовать der(10)t(3;10)(p25;p15). Цитогенетическое исследование (GTG-окрашивание хромосом) членов семьи показало, что мать, а также бабушка, тетя и сибс пробанда без клинических и фенотипических аномалий являются носителями сбалансированной хромосомной перестройки – t(3;10)(p25;p15). У сибса пробанда, девочки с тяжелыми когнитивными, неврологическими нарушениями и аномалиями развития, определен кариотип 46,ХХ,der(3)t(3;10)(p25;p15)dmat. Молекулярное кариотипирование позволило уточнить размер хромосомного дисбаланса и точки разрыва на обеих хромосомах, вовлеченных в транслокацию. В статье представлено описание клинико-фенотипических особенностей при наличии в кариотипе дериватных хромосом 3 и 10. Обсуждаются механизм формирования хромосомного дисбаланса у членов семьи с аномалиями фенотипа, зависимость тяжести клинических проявлений от размера и генного состава обнаруженных хромосомных перестроек, а также необходимые мероприятия, направленные на предупреждение рождения ребенка с хромосомной патологией в семьях носителей аутосомных реципрокных транслокаций.

## Introduction

Reciprocal translocations (RT) involve the reciprocal exchange of genetic material between two chromosomes, with a breakpoint occurring in each chromosome. Such exchanges can be balanced (if no chromosomal material is lost or gained) or unbalanced (if there is a net loss or gain of genetic material in one or both chromosomes). RT is one of the most common structural chromosomal abnormalities, with an estimated frequency of 1 in 500 to 1 in 625 newborns (Ogilvie, Scriven, 2002). The population frequency of balanced translocation carriers ranges from 0.08 to 0.3 % (Kochhar, Ghosh, 2013).

As a rule, carriers of reciprocal translocations (RT) are phenotypically normal. However, their reproductive potential is often compromised by an increased risk of infertility, recurrent miscarriage, or the birth of children with intellectual disabilities and multiple congenital anomalies. This risk arises from the high likelihood of chromosomal imbalance in the offspring, resulting from aberrant meiotic segregation in RT carriers (Hu et al., 2016).

Chromosome segregation patterns are established during meiosis I, and in rare cases, may also result from errors in meiosis II. In RT carriers, the formation of bivalents between non-homologous chromosomes involved in the translocation becomes impossible during prophase I. Instead, a quadrivalent structure forms, ensuring complete homosynapsis between the rearranged chromosomes. During gametogenesis, three segregation patterns are possible – 2:2, 3:1, and 4:0 – reflecting the distribution of chromosomes from the quadrivalent to daughter gametocytes. The predominant segregation pattern is largely determined by the quadrivalent configuration, which itself depends on the breakage–reunion points in the rearranged chromosomes (Gardner, Amor, 2018).

Of the 32 theoretically possible gamete combinations resulting from meiotic segregation in reciprocal translocation (RT) carriers, only two produce genetically balanced gametes: those containing either both non-rearranged chromosomes or both derivative chromosomes (alternate 2:2 segregation pattern). All the other segregation patterns result in gametes with chromosomal imbalance. The 2:2 malsegregation patterns include: adjacent-1 segregation – produces gametes with partial trisomy/monosomy of the translocated segment; adjacent-2 segregation – leads to partial trisomy/monosomy of the centric segment. In 3:1 segregation, gametes with 22 or 24 chromosomes are formed. Resulting zygotes contain 45 or 47 chromosomes. Zygotes with 47 chromosomes (trisomic) demonstrate the highest viability among unbalanced outcomes. In 4:0 segregation gametes receive either all four chromosomes or none from the quadrivalent. Resulting zygotes exhibit either double trisomies or double monosomies. These zygotes are uniformly nonviable (Shilova, 2016).

The viability of carriers and the severity of clinical manifestations in cases of chromosomal imbalances depend on three key factors: the size of the imbalanced region, its chromosomal location, specific genes involved in the affected regions. Notably, translocations with terminal breakpoints demonstrate a significantly increased frequency of embryos with chromosomal imbalance. The terminal location of breakpoints represents an independent risk factor resulting in the birth of viable offspring with multiple congenital anomalies, chromosomal imbalance. Statistical analysis reveals that carriers of RTs with at least one terminal breakpoint (0.2 of the size of the respective chromosome arm and less) have a 6-fold increased risk of producing viable offspring with these adverse outcomes compared to RTs without terminal breakpoints (Shilova, 2019). When chromosomal imbalances affect genes critical for embryonic development, developmental arrest typically occurs either during early embryogenesis or later in prenatal development (Beyer et al., 2019). In cases where the imbalance is compatible with continued in utero development, gestation typically results in the birth of a child with congenital malformations and/or developmental abnormalities (Shilova, 2016).

This study investigates the phenotypic and genetic consequences of meiotic segregation patterns in translocations between chromosomes 3 and 10, specifically involving their terminal regions, across three generations of a single family. We present: a clinical case of 3p deletion syndrome resulting from genomic imbalance in a female, with concurrent cases of 10p15 deletion syndrome in male and female cousins.

## Materials and methods

**Fig. 1.**
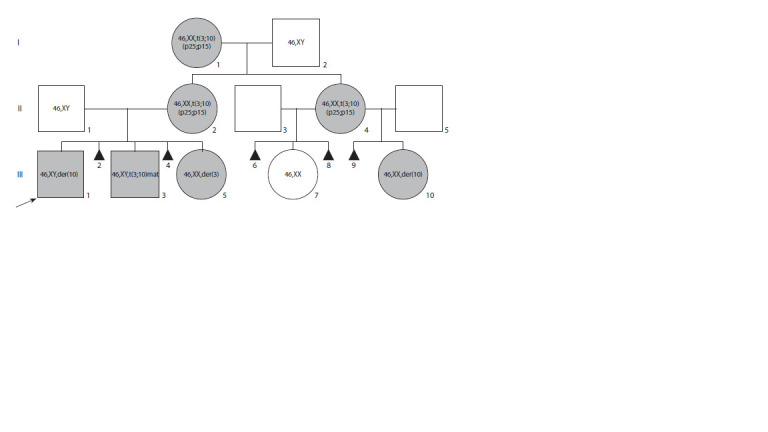
Schematic representation of one and the same family members – carriers of the translocation t(3;10)(p25;p15) – examined across three generations (the arrow points to the proband).

Proband III-1, a boy born in 2006, was first evaluated by a clinical geneticist at the Chelyabinsk Regional Children’s Clinical Hospital in 2009. In 2018, his newborn sister (III-5) and parents (II-1, II-2) underwent cytogenetic analysis. Six additional family members, including the proband’s brother (III-3), maternal aunt (II-4), her two daughters (III-7, III-10), as well as the maternal grandmother (I-1) and grandfather (I-2), were examined in 2024 (Fig. 1).

Cytogenetic analysis was performed on GTG-banded metaphase chromosome preparations (550-band resolution) obtained from PHA-stimulated peripheral blood T-lymphocytes, following a standardized cytogenetic protocol (Medical Genetics, 2022).Molecular and cytogenetic investigations were performed in an external laboratory. High-resolution chromosomal microarray analysis (CMA) was conducted using the Affymetrix CytoScan HD oligonucleotide microarray platform, following the manufacturer’s protocol (Affymetrix, USA). Data analysis was performed using the Chromosome Analysis Suite (ChAS) software (v4.0). Clinical exome sequencing (CES) was performed via next-generation sequencing (NGS) with paired-end reads. Sequencing data were processed by aligning reads to the human reference genome (GRCh38/hg38). The DECIPHER database was utilized to assess genes within the chromosomal imbalance region for haploinsufficiency and triplosensitivity effects.Cytogenetic and CMA results were interpreted according to the International System for Human Cytogenomic Nomenclature (ISCN 2024).

All studies involving human participants complied with the ethical guidelines of the National Committee for Research Ethics and the Declaration of Helsinki (1964, with later amendments). Written informed consent was obtained from all participants or their legal guardians

## Results

Male proband (III-1), born in 2006, was the product of an uncomplicated first pregnancy and delivery. His birth parameters included: weight: 2,600 g (1st–2nd centile, ~3 %), length: 51 cm (4th–5th centile, ~50 %), Apgar scores: 7/8. The boy exhibited significant psychomotor delay: head control achieved at 3 months, independent sitting at 10–11 months, ambulation at 2.5 years (developed progressively stiff gait). The first genetic assessment (2009) at Chelyabinsk Regional Children’s Clinical Hospital revealed normal male karyotype (46,XY) on GTG-cytogenetics. Despite recommendations for annual follow-up, the family was lost to genetic surveillance for 9 years.

The patient was re-evaluated at the same institution due to progressive neurological deterioration and admitted to neurology service (2023). At age 7, the patient experienced significant motor regression – lost ambulation capacity (currently only able to crawl), markedly limited expressive language (5-word vocabulary). A history of seizure-like episodes was characterized by ocular squeezing, risus sardonicus (sustained grimacing), perioral cyanosis, respiratory distress, myoclonic jerks of extremities. Clinical exome sequencing (CES) was performed in 2024 to investigate progressive neurodevelopmental regression, complex seizure disorder, suspected underlying genetic etiology.

CES revealed that proband III-1 carried a 2,993,266 bp deletion on the short arm of chromosome 10 (chr10:179763– 3173029), encompassing 27 genes, including 10 proteincoding genes. Among these, three were OMIM-annotated: ZMYND11 (associated with autosomal dominant intellectual developmental disorder 30 [AD]), WDR37 (linked to neurooculo-cardiogenitourinary syndrome [AD]), PITRM1 (implicated in autosomal recessive spinocerebellar ataxia 30 [AR]). Additionally, a 9,809,749 bp duplication was detected on the short arm of chromosome 3 (chr3:2735965–12545714), spanning 115 genes (58 protein-coding ones). These findings imply that the proband carries a derivative chromosome 10 resulting from a translocation between chromosomes 3 and 10.

In 2018, the proband’s parents sought medical genetic counseling for their three-month-old daughter (III-5). The girl was born from the mother’s (II-2) fifth pregnancy, which occurred against a background of complicated obstetric-gynecological history. This was the mother’s third delivery, resulting in a fullterm cesarean section. The mother’s previous pregnancies included: two live births – the proband (III-1, born in 2006) and a male sibling (III-3, born in 2012), two spontaneous abortions in early gestation (unknown etiology; no genetic or cytogenetic analysis was performed on the embryos). Prenatal findings in III-5 included marginal chorion presentation, intrauterine growth restriction (IUGR), congenital heart disease (CHD), musculoskeletal anomaly, polydactyly of the upper extremities. The gestation was complicated by chronic decompensated placental insufficiency and polyhydramnios. Birth parameters (III-5) were as follows: birth weight: 1,970 g (<1st centile, 3 %), length: 45 cm (1st centile, 3 %), head circumference: 35 cm (5th centile, 75 %), thoracic circumference: 30 cm (1st centile, 3 %). These anthropometric measurements indicate severe intrauterine growth restriction (IUGR). The Apgar score was 7. Clinical and phenotypic features included multiple congenital malformations, subclinical hyperthyroidism, iliac ectopia of the left kidney, flexion contractures of both thumbs, hip dysplasia, grade 2 cerebral ischemia, movement disorder suppression syndrome, ocular abnormalities: microphthalmia, microcornea. Congenital heart disease (CHD) included primum atrial septal defect (ASD), persistent left superior vena cava, patent foramen ovale (PFO), elongated Eustachian valve, grade 1 mitral regurgitation, group 1 pulmonary hypertension, circulatory failure (class 2A).

**Fig. 2.**
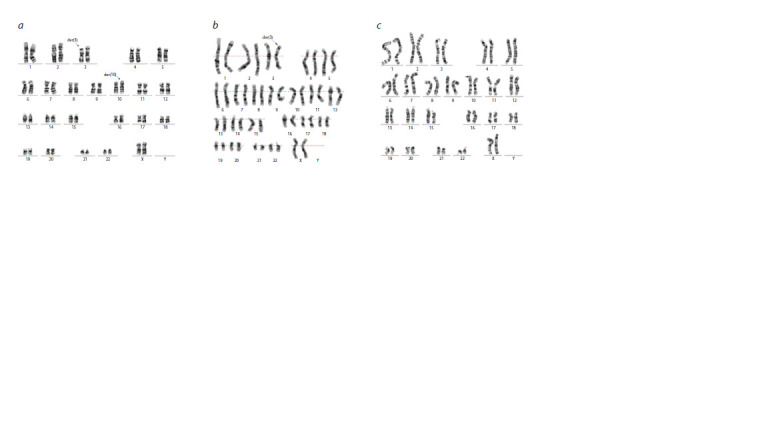
Unbalanced and balanced variants of familial translocation between chromosomes 3 and 10. a – karyotype of the mother II-2, reciprocal translocation t(3;10)(p25;p15); b – karyotype of the proband’s sister III-5 46,XX,der(3)t(3;10)(p25;p15)dmat; c – karyotype of the proband’s female cousin III-10 46,ХХ,der(10)t(3;10)(p25;p15)dmat). GTG-banding of the chromosomes.

The cytogenetic investigation revealed derivative chromosome 3, karyotype 46,XX,der(3). Parental karyotyping clarified the origin of chromosomal anomaly: the mother (II-2) is the carrier of a reciprocal translocation – 46,ХХ,t(3;10) (p25;p15). The father (II-1) had a normal male karyotype – 46,XY. Thus, the karyotype of the proband’s sister (III- 5) was determined as 46,XX,der(3)t(3;10)(p25;p15)dmat (Fig. 2).

Molecular karyotyping of the proband’s sister (III-5) precisely delineated the genomic imbalance and translocation breakpoints: arr[GRCh38] 3p26.3p25.2(11007_12547742) x1,10p15.3p15.2(45908_3500569)x3. Chromosome 3 deletion can be described as follows: size: 12,536,735 bp, genes affected: 132 (60 protein-coding ones), including 37 OMIMannotated genes. Chromosome 10 microduplication was 3,454,661 bp in size, genes affected: 33 (11 protein-coding ones).

**Fig. 3.**
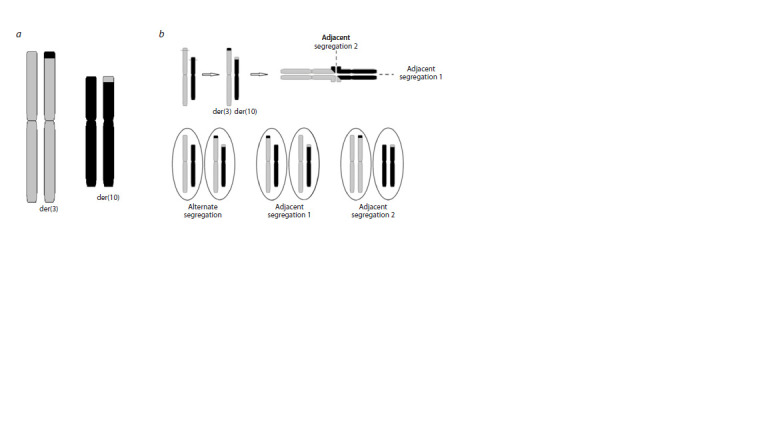
Schematic representation of: а – reciprocal translocation formation between chromosomes 3 and 10, t(3;10)(p25;p15); b – variants of meiotic segregation patterns of chromosomes with translocation.

It can be concluded that the chromosomal imbalances observed in proband III-1 (karyotype: 46,XY,der(10)t(3;10) (p25;p15)mat), who inherited the derivative chromosome 10, and sibling III- 5, who inherited the derivative chromosome 3, result from meiotic adjacent-1 2:2 malsegregation of a maternal reciprocal translocation t(3;10)(p25;p15) (Fig. 3).

In 2024, the proband’s parents sought genetic re-evaluation for their 12-year-old son (III-3) (born 2012), who exhibited no clinical abnormalities. G-banding revealed a balanced translocation, 46,XY,t(3;10)(p25;p15)mat, inherited from his mother (II-2).

The same year, the mother’s sister (II-4) consulted medical geneticist with her two daughters: a phenotypically normal older daughter (III-7, born 2006) (46,XX) and a younger daughter (III-10, born 2017) presented with delayed motor development (gait instability, limited speech) and dysmorphic features. The karyotype of the younger girl showed an unbalanced derivative chromosome 10: 46,XX,der(10)t(3;10) (p25;p15)mat. Molecular karyotyping of the mother (II-2) revealed a balanced reciprocal translocation t(3;10)(p25;p15). Parental karyotyping traced the translocation to the grandmother (I-1), confirming a familial balanced translocation, 46,XX,t(3;10)(p25;p15) (Fig. 1).

Thus, in the presented family the daughters II-2, II-4 and the grandson III-3 inherited balanced translocation 46,XX,t(3;10) (p25;p15) from the grandmother I-1. As a result of meiotic adjacent-1 malsegregation proband III-1 and his cousin III- 10 had unbalanced karyotype with derivative chromosome der(10) and the karyotype of proband’s sister III-5 with the severe clinical manifestations contains derivative chromosome der(3) (Fig. 3).

## Discussion

Chromosome banding analysis at a 550-band resolution per haploid set enables the detection of chromosomal abnormalities in 3–10 % of patients with intellectual disability. GTG-banding remains the gold standard for diagnosing chromosomal imbalances exceeding 8–10 Mb in size (Lebedev et al., 2023). However, as with any method relying on subjective interpretation, the accuracy of results depends heavily on the cytogeneticist’s expertise, chromosome spreading quality, and banding clarity. Unfortunately, the derivative chromosome 10 (der(10)) in the proband (III-1) was not detected during initial cytogenetic analysis in 2009, significantly prolonging the “diagnostic odyssey” of this family.

As shown in Figures 1 and 3, four family members exhibited a balanced/normal karyotype, resulting from maternal alternate meiotic segregation of a familial reciprocal translocation t(3;10). In contrast, three individuals – the proband (III-1) and his cousin (III-10) (both with der(10)), as well as the proband’s sister (III-5, with der(3)) – had unbalanced karyotypes due to adjacent-1 malsegregation (2:2 segregation). This suggests that balanced and unbalanced gametes (zygotes) are formed at near-equal frequencies (4:3) during meiotic segregation of this translocation. Literature analysis indicates that meiotic malsegregation occurs in ~30 % of reciprocal translocation cases, with adjacent-1 segregation being the most prevalent (~80 %). Adjacent-2 segregation is observed in ~13 % of cases, while tertiary (exchange) segregation accounts for ~7 % (Shilova, 2016).

Given that both mothers (II-2 and II-4) had a history of spontaneous abortions (the grandmother’s obstetric history is unknown), we hypothesize that these pregnancy losses may represent embryos with chromosomal imbalances resulting from either meiotic adjacent-2 segregation of the familial translocation, or aneuploidy involving chromosomes 10 and 3 (which would be incompatible with live birth).

Presumably, in this reciprocal translocation, only conceptuses with chromosomal imbalances arising from adjacent-1 segregation (2:2) appear viable. Literature evidence suggests that when breakpoints occur in terminal chromosomal regions (as in our case), the likelihood of live-born children with multiple congenital anomalies and/or chromosomal imbalances increases sixfold (Shilova et al., 2019). In the present study, both breakpoints were located in terminal regions, which likely contributed to the birth of affected children with severe phenotypic manifestations

The limited family pedigree does not permit a statistically robust assessment of the empirical recurrence risk for chromosomal imbalance in offspring. Nevertheless, our findings indicate a substantial observed risk (3/7, or 43 %) of unbalanced outcomes. This information is particularly relevant for the proband’s brother (III-3), a balanced carrier of t(3;10), and should inform both clinical counseling and his future reproductive planning. Notably, among the grandmother’s five grandchildren, only III-3 and one cousin (III-7) show no clinically significant phenotypic abnormalities. The other three (III-1, III-5, and III-10) exhibit developmental defects and multiple congenital anomalies resulting from genomic imbalance. While modern reproductive technologies (such as PGD) could mitigate this risk, the high probability of unbalanced segregation warrants heightened clinical vigilance regarding the reproductive choices of translocation carrier III-3. For instance, longitudinal follow-up of families with identified chromosomal rearrangements (diagnosed 15–34 years prior) revealed two significant findings: in four families, parents had no recollection of their children’s previous cytogenetic diagnoses, and in four additional families (representing approximately 10 % of the study cohort), parents failed to comprehend the clinical implications of the karyotyping results (Bache et al., 2007).

Molecular characterization using chromosomal microarray analysis (CMA) and clinical exome sequencing (CES) in the proband (III-1) and his affected sister (III-5) enabled precise mapping of translocation breakpoints, identification of genes contained within the unbalanced chromosomal regions. Based on the karyotyping results, we hypothesized that the proband (III-1) and his female cousin (III-10) carried a genomic imbalance involving a 10р15-pter deletion and a 3р25-pter duplication, indicating the presence of a derivative chromosome 10 (der(10)). In contrast, chromosomal microarray analysis (CMA) of the proband’s sister (III-5) identified a derivative chromosome 3 (der(3)) with a terminal 3р25-pter deletion (12.5 Mb) and a 10р15-pter duplication (3.5 Mb) (see the Table).

**Table 1.**
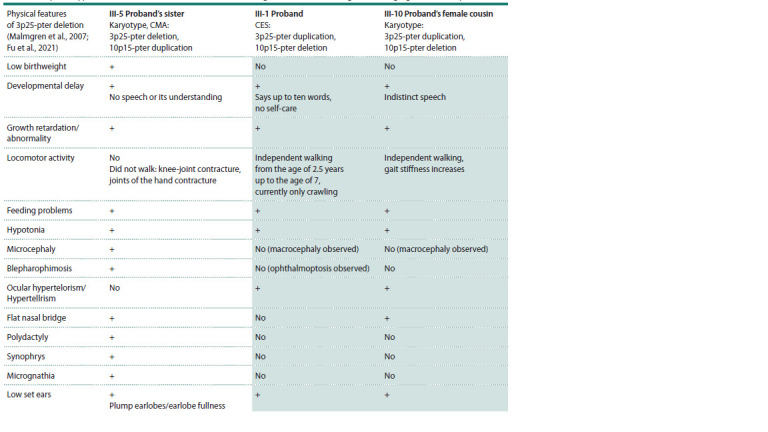
Clinical and phenotypic manifestations in children with unbalanced genome formed during meiotic segregation t(3;10)(p25;15) Notе. Children with similar chromosomal imbalance are marked with a background.

Although all patients with chromosomal imbalances in our cohort exhibited severe cognitive and physical impairments, detailed clinical evaluation revealed that the phenotypes of the two der(10) carriers were similar but different from that of the proband’s sister (III-5) with der(3).

The terminal deletion of the 10p15-pter region was first reported by D. Elliott et al. (1970). To date, approximately 50 cases of 10p15-pter deletions with varying lengths have been described in the literature. The core clinical features associated with 10p15-pter deletions include cognitive impairment, behavioral abnormalities, speech delay, locomotor dysfunction, craniofacial dysmorphism, hypotonia, brain malformations, and seizures. These features are attributed to haploinsufficiency of the ZMYND11 (OMIM 608668) and DIP2C (OMIM 611380) genes (DeScipio et al., 2012); both are located within the deleted region identified in proband III- 1 and his female cousin III-10, who exhibited characteristic clinical manifestations. Notably, the phenotypic spectrum in these cases may reflect not only the 10p deletion but also the concurrent 3p25-pter duplication, which encompasses 13 protein-coding genes known to be triplosensitive. The patients require specialized neurorehabilitation, particularly the female cousin III-10, who demonstrates progressive locomotor deterioration similar to proband III-1.

In contrast, patient III-5 (the proband’s sister) exhibited severe clinical features consistent with 3p deletion syndrome, further supporting the pathogenicity of the 3p25-pter deletion identified in her case (Verjaal, De Nef, 1978; Malmgren et al., 2007; Fu et al., 2021) (see the Table). Deletions of the terminal 3p region represent a rare chromosomal abnormality associated with characteristic phenotypic features, including microcephaly, ptosis, hypertelorism, and micrognathia. Affected individuals typically exhibit low birth weight, hypotonia, intellectual disability, developmental delay, delayed bone maturation, and renal anomalies. Congenital heart defects – particularly atrioventricular septal defects – occur in approximately one-third of cases (Martins et al., 2021). In our study, the proband’s sister (III-5) had severe manifestations from birth, including low birth weight, complete absence of locomotor activity and speech, microcephaly, polydactyly, synophrys, and micrognathia. Notably, these features were absent in the proband (III-1) and his female cousin (III-10), highlighting the phenotypic divergence between the two genomic imbalances. Of particular interest is the contrasting cranial growth patterns observed: microcephaly in III-5 (with 3p25-pter deletion) versus macrocephaly in cases with 3p25- pter duplication. This reciprocal phenotype likely reflects dosage sensitivity of genes within this region, underscoring the critical role of gene copy number in neurodevelopment and craniofacial morphogenesis.

As previously noted, the 3p25-pter deletion region encompasses 132 genes, including 25 morbid genes associated with clinical phenotypes. Among these, haploinsufficiency of SETD5, BRPF1, CRBN, ATG7, SLC6A11, GRM7, and ARPC4 has been linked to neurodevelopmental disorders and cognitive impairment. Notably, the region also includes CHL1, a candidate gene for nonspecific intellectual disability due to its high expression in the developing brain (Martins et al., 2021; Tsuboyama, Iqbal, 2021). The concurrent 10p15-pter duplication in this patient may further contribute to the abnormal phenotype, as this region contains two triplosensitive genes: LARP4B and DIP2C. Of these, DIP2C has been specifically associated with developmental delay and speech impairment, suggesting a potential additive or synergistic effect of the dual genomic imbalance

## Conclusion

The 14-year clinical odyssey of this three-generation family enabled the identification of carriers with both the balanced reciprocal translocation t(3;10) and derivative chromosomes resulting from adjacent-1 meiotic 2:2 malsegregation. Breakpoints were precisely mapped using high-resolution genomic techniques – chromosomal microarray analysis (CMA) and clinical exome sequencing (CES). Variability in the size and gene content of the imbalanced regions correlated with the severity of phenotypic abnormalities among affected individuals. Notably, patients sharing similar genomic imbalances exhibited comparable and progressively worsening clinical manifestations, underscoring the necessity for multidisciplinary care involving neurologists and rehabilitation specialists.

Chromosomal imbalances involving concurrent terminal deletions and duplications of non-homologous chromosomes typically arise from meiotic segregation errors in parental reciprocal translocations. Thus, parental karyotyping is essential to identify translocation carriers. Although the proband’s brother (III-3) with the balanced translocation remains asymptomatic, genetic counseling is critical to inform him of his reproductive risks. Advanced preimplantation (PGT) and prenatal diagnostic methods can significantly reduce the likelihood of transmitting severe genomic disorders to his offspring.

## Conflict of interest

The authors declare no conflict of interest.
